# Complete Genome Sequences of Nine Phages Capable of Infecting *Paenibacillus larvae*, the Causative Agent of American Foulbrood Disease in Honeybees

**DOI:** 10.1128/genomeA.01120-15

**Published:** 2015-10-15

**Authors:** Philippos K. Tsourkas, Diane G. Yost, Andrew Krohn, Lucy LeBlanc, Anna Zhang, Casey Stamereilers, Penny S. Amy

**Affiliations:** a School of Life Sciences, University of Nevada, Las Vegas, Nevada, USA; b Biological Sciences, Northern Arizona University, Flagstaff, Arizona, USA; c University of Rochester, Rochester, New York, USA

## Abstract

We present here the complete genome sequences of nine phages that infect *Paenibacillus larvae*, the causative agent of American foulbrood disease in honeybees. The phages were isolated from soil, propolis, and infected bees from three U.S. states. This is the largest number of *P. larvae* phage genomes sequenced in a single publication to date.

## GENOME ANNOUNCEMENT

American foulbrood disease, caused by the bacterium *Paenibacillus larvae*, is the most destructive bacterial disease affecting the honeybee, *Apis mellifera* (1). Its strains are rapidly becoming antibiotic resistant (2), and infected colonies must be burned in order to control the spread of the bacterium (3). Phages that infect and lyse *P. larvae* are a potentially promising treatment, but they have only recently begun to be characterized. There are currently seven complete *P. larvae* phage genome sequences in the literature (4, 5). Here, we have isolated and sequenced the genomes of nine *P. larvae* phages obtained from samples across the United States.

Samples were collected from soil near beehives, propolis, cosmetics containing beeswax or royal jelly, infected larvae, and phages induced from lysogeny in *P. larvae* strains. The environmental samples came from Nevada, Maryland, and Washington state. Phages were amplified using *P. larvae* NRRL 2605, an enterobacterial repetitive intergenic consensus (ERIC) I genotype strain, and plated on modified brain heart infusion agar with soft agar overlays (6). DNA was purified using either the Qiagen DNeasy or Norgen phage DNA isolation kit. One nanogram of DNA per sample was used to produce 49 random-sequencing libraries, using the Nextera XT DNA sample preparation kit, which were then sequenced on a MiSeq 50 desktop sequencer.

The distance between paired-end reads was set to either 400 or 500 bp. The reads were assembled into contigs using Geneious version 7.1 using medium/fast sensitivity, disallowing gaps. The assembly process for phages Fern, Harrison, Paisley, Willow, and Xenia produced complete genomes. This was not the case for phages Diane, Hayley, Vadim, and Vegas, so for these phages, PCR probes were designed to begin 600 bp downstream of the contig start and 300 bp upstream of the contig end. The PCR amplicons were then spliced into the contig to produce the complete genome. All nine phages are *Siphoviridae* with linear double-stranded DNA (dsDNA) genomes. The DNA packaging strategy was identified as “cohesive ends with 3′ overhangs” (7). The overhangs are CGACTGCCC for phages Diane, Fern, Hayley, Vadim, Vegas, Willow, and Xenia, and CGACGGACC for phages Harrison and Paisley. The genomes were rearranged by setting the first base of the genome to be the base immediately after the 3′ overhang.

The genomes were annotated using DNA Master. The criteria used to determine the validity of gene calls include autoannotation calls by Glimmer, GeneMark, and GeneMark.hmm, the coding potential map produced by GeneMark.hmm using *Paenibacillus polymyxa* SC2 as the reference strain; gene length criteria (with calls <150 bp, 120 bp, and 90 bp treated with increasing skepticism); BLAST results with *E* value <0.001; Shine-Dalgarno sequence of >200 nucleotides (nt) using the Old DNA Master scoring method; and whether the gene call significantly overlapped (>30 bp) other gene calls. Preliminary analysis shows that phages Diane, Vadim, Vegas, and Hayley are very closely related to each other, as are phages Fern and Willow and phages Harrison and Paisley. The assembly and annotation results are shown in Table 1. Future studies will provide a detailed comparative genomic analysis of these and other *P. larvae* phages.

**TABLE 1:** *Paenibacillus larvae* phages, GenBank accession numbers, and genome assembly results

Phage name	GenBank accession no.	Genome length (bp)	Avg coverage depth (×)	G+C content (%)	No. of genes
Diane	KT361657	45,653	67	43.7	86
Fern	KT361649	37,995	502	41.9	68
Harrison	KT361651	44,247	291	40.2	84
Hayley	KT361655	44,256	43	43.5	84
Paisley	KT361653	44,172	350	40.0	84
Vadim	KT361656	45,653	94	43.7	86
Vegas	KT361654	45,653	128	43.7	86
Willow	KT361650	37,994	122	41.9	68
Xenia	KT361652	41,149	123	41.5	77

### Nucleotide sequence accession numbers.

The GenBank accession numbers are listed in Table 1.
